# Editorial: Optical coherence tomography and OCT angiography examination in systemic diseases

**DOI:** 10.3389/fmed.2023.1147909

**Published:** 2023-02-09

**Authors:** Livio Vitiello, Maddalena De Bernardo, Palmiro Cornetta

**Affiliations:** ^1^Eye Unit, “Luigi Curto” Hospital, Azienda Sanitaria Locale Salerno, Salerno, Italy; ^2^Eye Unit, Department of Medicine, Surgery and Dentistry, “Scuola Medica Salernitana”, University of Salerno, Salerno, Italy; ^3^Eye Unit, “Maria SS Addolorata” Hospital, Azienda Sanitaria Locale Salerno, Salerno, Italy

**Keywords:** OCT, optical coherence tomography, systemic disease, OCT angiography, OCTA

In recent years, optical coherence tomography (OCT) and optical coherence tomography angiography (OCTA) have been increasingly used not only to assess ocular diseases (1), but also to better evaluate systemic diseases, looking for possible ocular manifestations.

In fact, several studies have been published dealing with the use of these diagnostic tools to evaluate retinal and choroidal aspect in patients with systemic diseases, such as celiac disease (2, 3), and some neurological disorders, such as multisystemic atrophy (4) and progressive supranuclear palsy (5).

Furthermore, these imaging devices have also been used to monitor the effects on the ocular tissues of some systemic therapies, as in the case of alpha-lytic therapy administered to treat benign prostatic hypertrophy (6).

In this Research Topic four original research articles, one case report, one mini review, and one systematic review and meta-analysis focused on this challenging topic, highlighting the utility of these ocular diagnostic methods to provide useful information on potential ocular involvement in systemic diseases.

Chiosi et al. evaluated 152 patients positive for SARS-CoV-2 infection utilizing OCTA and compared them with a control group. They found the mean vessel density of deep capillary plexus and the choriocapillaris to be significantly lower in the foveal zone of the COVID-19 group. Therefore, the authors suggested a possible microvascular retinal impairment in these patients, also considering the potential hypercoagulable state assumed in the COVID-19 pathophysiology.

In their study, Montorio et al. appraised the possible correlation between the foveal avascular zone area and retinal vessel density in the near peripheral retina utilizing a wide-field OCTA in patients affected by relapsing-remitting multiple sclerosis, showing a reduced perfusion status in the near peripheral retina of multiple sclerosis patients. For this reason, the authors suggested a vascular dysfunction as a potential factor involved in the pathogenetic mechanisms of this neurological disease.

Coppolino et al. carried out a pilot study to analyze OCTA metrics in 15 patients at specific time-points during a hemodialysis session to better understand how these metrics gradually change and to assess potential correlations with patients' characteristics. The authors did not notice a significant variation of OCTA metrics, but they characterized the different effect of hemodialysis on retinal and choroidal circulation, also suggesting the possible role of autonomic system on vessel control in patients affected by uremia.

A very interesting study by Fu et al. was also included in this Research Topic, in which the authors used anterior segment OCT and confocal microscopy to better investigate a group of children with an R124L mutation corneal dystrophy. The OCT examination showed uneven corneal epithelial thickness, with the presence of abnormal substances in the Bowman's layer and corneal deposits becoming increasingly thicker. This study demonstrated OCT as a convenient, quick, and non-contact tool for screening and monitoring the pathological process of corneal dystrophies.

In their case report, Zhou et al. evaluated a 45-year-old man affected by pulmonary arterial hypertension and presented with decreasing central vision and metamorphopsia in both eyes. Utilizing OCTA, the authors demonstrated central serous chorioretinopathy-like abnormalities, maybe induced by the choroid congestion related to this pulmonary disease.

Finally, in the two review articles, Courtie et al. and Jiang et al. showed the usefulness of OCTA and OCT parameters as possible and valuable markers in case of sepsis and diabetes, respectively.

A correlation between axial length and choroidal thickness has been shown. Unfortunately, these papers did not consider that axial length are not very precise and need some corrections (7, 8). For this reason, to confirm this finding, further studies taking into consideration this correction would be advisable.

In conclusion, OCT and OCTA are gaining an increasingly important and relevant role in everyday clinical practice, not only for the evaluation of ocular diseases but also for more thoroughly investigating possible ocular involvement in different systemic diseases.

In the coming years, we suppose an increasing number of scientific papers will be published, in which these imaging devices will be used to better understand the underlying pathophysiology of various systemic diseases, and to monitor the possible effects of systemic therapies on the eye.

## Author contributions

All authors listed have made a substantial, direct, and intellectual contribution to the work and approved it for publication.
